# Ophthalmology

**Published:** 1905-09

**Authors:** C. H. Walker


### OPHTHALMOLOGY.

The operation of **excision of the eyeball** was practised with but little alteration from 1840 until 1885, when Mules suggested the evisceration of the eyeball and the introduction of a glass ball into the sclerotic. Frost's suggestion of excising the eye in the usual way, and inserting the ball into Tenon's capsule, was a great improvement, but was by no means invariably successful. In a fair proportion of the cases the ball was either not retained or caused some irritation.

As a substitute for the glass ball used by Frost in his modification of Mules's operation, Spratt[1] has recently used a paraffin sphere with success. Various substitutes for the glass ball have been tried, such as metal, vulcanite, and celluloid. Hard

[1] *Arch. Ophth.*, 1905, XXXIV. 123.

paraffin presents obvious advantages—it is cheap, it can be cut to any size, it cannot be broken, and it is easily held in forceps, so that no special "introducer" is necessary. The globe may be prepared in the following way:—Hard paraffin is melted and run into a test tube, and then sterilized in the usual way. When the paraffin has set the tube is heated slightly till the cylinder of paraffin can be slid into an antiseptic lotion. Balls of the required size are then carved. About 17 mm. is the most convenient size. The technique of Spratt's operation differs but little from Frost's, the essential thing being to suture the four recti over the ball with chromicised catgut. Tenon's capsule is similarly treated, and then the conjunctiva is closed over the two inner layers with a purse-string suture.

**Trans-illumination of the sclerotic** as an aid to the diagnosis of intra-ocular tumours has been re-introduced with the advent of Professor Leber's instrument. This consists of an 8 or 10 volt lamp in a small closed case, held by a short handle. A glass rod, about an inch long, abuts against the lamp bulb; its other end, which is sheathed in vulcanite, projects from the metal case. When the end of this rod is applied to the sclerotic of the cocainised eye no light escapes laterally owing to the vulcanite sheath, but a considerable amount of light penetrates the sclerotic, and the observer at once sees the pupil illuminated with a red glow. This is easily perceptible, even if there is a ripe cataract, but if the instrument is applied over the area occupied by an intra-ocular tumour little or no illumination of the pupil is seen. The instrument is chiefly of use in those cases where it is sought to diagnose between a simple detachment of the retina and one due to a sarcoma of the choroid. It is a very great improvement on some of the previous trans-illuminators, in which a small lamp had to be kept cool by means of a "water-jacket," with a glass pane in one side which was applied to the sclerotic. Leber's apparatus gets hot after a time and must be rested, but there is usually time to examine the eye thoroughly before the end of the rod gets too hot to be borne by the patient. Swanzy[1] gives details of three cases in which it was conspicuously useful as an aid to diagnosis.

**The healing of incisions in the cornea** differs essentially from the healing of "skin" incisions. Experiments on rabbits[2] show that in from five to fifteen minutes the wound is closed by a plug of fibrin which spreads out posteriorly into a fan- or mushroom-like expansion in the anterior chamber. This plug has its origin, not from the cut edges of the wound, but from the freshly secreted aqueous, which is highly albuminous. An hour after the operation the corneal epithelium begins to grow down into the wound. It is not mechanically carried into the wound

[1] *Ophth. Rev.*, Feb., 1905, p. 33. [2] *Arch. Ophth.*, 1905, xxxiv. 37.

with the knife, as Ranvier thought. There is no leucocyte infiltration whatever. In three or four hours spindle-shaped cells appear in the substantia propria and spread into the wound. On the fourth day the spindle cells in the wound have increased, and from these cicatricial tissue is eventually formed which presses out the ingrowth of epithelium. Considerable trace of epithelial ingrowth can be found for some months. It is a case of regeneration of connective tissue from its own elements. The plug of fibrin takes no active part in the regeneration. No trace of renewal of Descemet's membrane is visible for about a month after the operation, and its normal thickness is not acquired till after about four months. This delay is probably attributable to Descemet's membrane being a "hyalinisation" of the posterior layers of the substantia propria of the cornea and of the cicatricial tissue in the wound, the gradual result of its constant contact with the aqueous humour which reaches it through the stomata in the endothelial lining of the anterior chamber. Many of these facts are borne out by clinical and pathological investigation. Eyes lost after cataract extraction show pretty nearly the above changes in the incision, but in severe cases of irido-cyclitis there is much leucocyte infiltration in the posterior half of the corneal wound.

**Traumatic keratitis in the new-born.** Amongst the first to draw attention to this condition were Thomson and Buchanan, in the *Transactions of the Ophthalmological Society, 1902 and 1903.* Since then they have collected nine more cases,[1] most of them from the Glasgow Maternity Hospital, at which institution a great number of rickety dwarfs are delivered. Sydney Stephenson records two more cases, one met with at the Evelina Hospital, the other occurred at Queen Charlotte's Hospital. Thomson and Buchanan attribute the frequency of these cases in Glasgow to the fact that Glasgow is notorious for rickets, and consequently for contracted pelves. The recorded cases show an agreement in the following particulars:—1st, nearly all have followed a difficult instrumental delivery; 2nd, generally only one eye is affected; 3rd, they are associated with other injuries — abrasions, bruises, sub-conjunctival hemorrhages, hyphæma, &c.; 4th, that a more or less characteristic form of corneal opacity is usually present. There are two forms, a temporary diffuse opacity and a permanent linear opacity. The first form is the more frequent, is due to œdema of the cornea, and passes off in a few days. The second form is due to rupture of the posterior elastic lamina of the cornea. The rupture is usually more or less vertical; sometimes there are two or more parallel ruptures. The grey streak left after the general œdema subsides gradually gets fainter, but with the pupil dilated it is visible for many months, and is probably

1 *Ophthalmoscope*, June, 1905.

permanent. Besides the linear opacity which persists there is likely to be, in most cases, considerable refractive error, and it is probable that these lesions account for a good many cases of monocular astigmatism.

**Interstitial keratitis** is relatively common in Bristol, and its intimate association with an injury to the eye must not be lost sight of. Text-books say little or nothing on the subject of injury being a determining factor, although many clinical observers are well aware of the fact. We have seen several such cases, and it is important to remember that a guarded prognosis must be given in all cases of injury where the patient suffers from congenital syphilis, more especially if he is out of health at the time. It seems possible that a good many cases of interstitial keratitis might be avoided altogether if this liability were borne in mind, and proper care and protection were afforded to what might appear to be trivial injuries. Why interstitial keratitis should in such cases also affect the other eye cannot be explained, but in non-traumatic cases the inflammation in the second eye is frequently more severe than in the first-affected eye, in spite of the fact that the patient has been under appropriate treatment all the time. It would appear from this that in nearly all specific cases an attack in the second eye is practically inevitable.

**The danger of using adrenalin** too freely in ophthalmic practice is not sufficiently recognised. Experimentally it has been shown that the first effect of instillation of adrenalin is diminution of the intra-ocular tension due to constriction of the ciliary vessels and consequent decreased secretion, followed by a slight increase of tension. Where there is no predisposition to glaucoma this result involves no risk whatever. MacCallan[1] was among the first to report cases where adrenalin acted harmfully in glaucoma. In five instances the tension rapidly increased after its application. It is claimed by some, however, that if the instillation is repeated at intervals of half an hour regularly vaso-constriction is maintained and permanent reduction of tension is secured. But it must be stated that Grand Clément, who advocates this method, uses eserine in conjunction with adrenalin, and that he only uses it in recent glaucoma, which considerably discounts its therapeutic value. It appears to be particularly liable to act injuriously in cases of hemorrhagic glaucoma, and we have recently seen a case in which its action was unquestionably harmful. The physiological action of different preparations of adrenalin varies considerably, and some eyes are much more profoundly affected by the drug than are others. The ordinary strength of 1-1000 is certainly unnecessarily strong for all cases, except perhaps operations,

[1] *Tr Ophth. Soc. U. Kingdom*, 1903, xxiii. 374.

and it would be a good rule in all doubtful cases to commence with a 1-10,000 solution.

**The Services and colour blindness.**—On the 10th of April Mr. Arnold-Forster stated that in accordance with the recommendations of the medical authorities, it had been decided to return to the former practice under which colour blindness was not regarded as a disqualification for a commission in the army.[1] It is to be hoped that after this most sensible concession the other regulations as to eyesight in the army will be framed on a more rational basis. At present a candidate with a considerable degree of hypermetropia may be accepted, when it would be safe to predict that in a very few years spectacles would become an absolute necessity. The importance of recognising visual defects is far greater, of course, in the navy and mercantile marine. In June, 1904, the P. & O. steamer *Australia* was wrecked at the entrance to Port Philip Harbour.[2] The pilot was found to have 3.0 D. myopia and some astigmatism. His colour vision as tested by Holmgren's wools was normal, but "tested by luminous discs considerable illumination was required to elicit a satisfactory discrimination of some colours." A point of considerable interest is that *the same pilot was in charge* when, on March 10th, 1904, another steamship was wrecked at the entrance to the harbour! The Marine Board of Victoria has made the following regulation for pilots since this accident:—"That the vision must be $\frac{6}{6}$ in each eye without glasses, and that the error of refraction must not exceed 1 D., tested under a mydriatic: colour vision is to be normal." It appears probable that the pilot in question had, as well as the myopia, some "shortening of the spectrum," that is to say, inability to appreciate the ends of the spectrum properly. Inability to appreciate the rays of lowest refrangibility at the red end would not be shown by Holmgren's wool test. This test, according to Edgridge-Green, is inaccurate as applied by the Board of Trade representatives. According to him, about 40 per cent. of those who appealed against their rejection were found to have been wrongly excluded. The "lantern test,"[3] devised by Edridge-Green, has the advantage of detecting those who have a shortened red spectrum. A lantern is provided with thirteen slides, seven of coloured glass and the rest modifying glasses. The chief feature is the use of two red slides, which look alike to the normal sighted, but one of which spectroscopically is both red and green, and therefore is seen as green by those who have much shortening of the red end of the spectrum.

C. H. WALKER.

[1] *Brit. M. J.*, 1905, i. 846. [2] *Lancet*, 1904, ii. 1202.
[3] *Tr. Ophth. Soc. U. Kingdom*, 1903, xxiii. 206.

